# Hydraulic Fracture Propagation in Topological Fractured Rock Masses: Insights from Visualized Experiments and Discrete Element Simulation

**DOI:** 10.3390/ma19010025

**Published:** 2025-12-20

**Authors:** Xin Gong, Jinquan Xing, Cheng Zhao, Haoyu Pan, Huiguan Chen, Jialun Niu, Yimeng Zhou

**Affiliations:** 1 College of Civil Engineering, Tongji University, Shanghai 200092, China; gongxin1015@163.com (X.G.); zhaocheng@tongji.edu.cn (C.Z.); panhaoyu@tongji.edu.cn (H.P.); chenhuiguan@tongji.edu.cn (H.C.); niujialun@tongji.edu.cn (J.N.); 2 School of Architectural Engineering, Jinggangshan University, Ji’an 343009, China; 3 Key Laboratory of Geotechnical and Underground Engineering of Ministry of Education, Tongji University, Shanghai 200092, China; 4 College of Engineering, Tibet University, No. 10, Zangda East Road, Chengguan District, Lhasa 850001, China; 5 China Construction Sci-Tech Innovation Group Co., Ltd., 869 Zhonghua Road, Huangpu District, Shanghai 200010, China; zhouyimeng@cscec.com

**Keywords:** fractured rock mass, topological structure, hydraulic fracturing, Digital Image Correlation, Discrete Element Method

## Abstract

**Highlights:**

**What are the main findings?**

- Hydraulic cracks initiate preferentially at tips closest to the loading boundary.
- Fracture topology plays a dual role: overall damage-weakening versus local configuration-strengthening.
- Topological fracture controls crack initiation by regulating stress concentration patterns.

**What are the implications of the main findings?**

- They provide a new topological perspective on fracture behavior in fracture network.
- They offer insights for optimizing fracturing designs in engineered materials and systems.
- The topology-based characterization method is a potential predictive tool.

**Abstract:**

The topological structure of fracture networks fundamentally controls the mechanical behavior and fluid-driven failure of brittle materials. However, a systematic understanding of how topology dictates hydraulic fracture propagation remains limited. This study conducted experimental investigations on granite specimens containing 10 different topological fracture structures using a self-developed visual hydraulic fracturing test system and an improved Digital Image Correlation (DIC) method. It systematically revealed the macroscopic control laws of topological nodes on crack initiation, propagation path, and peak pressure. The experimental results indicate that hydraulic crack initiation follows the “proximal-to-loading-end priority” rule. Macroscopically, the breakdown pressure shows a significant negative correlation with topological parameters (number of nodes, number of branches, normalized total fracture length). However, specific configurations (e.g., X-shaped nodes) can exhibit a configuration-strengthening effect due to dispersed stress concentration, leading to a higher breakdown pressure than simpler topological configurations. Discrete Element Method (DEM) simulations revealed the underlying mechanical essence at the meso-scale: the topological structure governs crack initiation behavior and initiation pressure by regulating the distribution of force chains and the mode of stress concentration within the rock mass. These findings advance the fundamental understanding of fracture–topology–property relationships in rock masses and provide insights for optimizing fluid-driven fracturing processes in engineered materials and reservoirs.

## 1. Introduction

The safety and efficiency of deep engineering projects, such as unconventional resource extraction and enhanced geothermal systems, are fundamentally controlled by the mechanical and fluid transport properties of the involved materials. These properties, in turn, are dominantly influenced by the internal fracture networks within rock masses [1]. In complex environments subject to tectonic movements and engineering disturbances, these discontinuous structural planes interweave. They dominate the mechanical strength, deformation characteristics, and fluid seepage behavior of the rock mass [2,3]. Hydro-mechanical coupling represents a core challenge in deep rock mass engineering. Specifically, the presence and seepage of groundwater alter the effective stress state, thereby affecting the evolution of fracture networks. Conversely, in active processes like hydraulic fracturing, fluid pressure directly drives fracture initiation and propagation [4,5]. Natural fracture networks can weaken the overall strength of the rock mass and accelerate cooperative rupture and connection between topological branches [6]. Engineering practice repeatedly confirms their decisive influence. For instance, the efficiency of hydraulic fracturing in shale gas reservoirs depends strongly on how natural fracture networks guide the flow paths of fracturing fluid [7,8]. Meanwhile, water inrush disasters in deep buried chambers are often linked to hydraulically connected channels controlled by specific topological structures [9]. Therefore, deeply understanding the control mechanisms of fracture networks, particularly their topological structure, on hydraulic fracture propagation is of crucial theoretical and practical significance for predicting rock mass behavior, optimizing engineering design, and preventing geotechnical hazards.

Accurate characterization of fracture networks is the foundation for understanding their behavior. Early research primarily focused on the geometric parameters of single fractures (e.g., trace length, dip angle, spacing) [10,11]. As research deepened, scholars developed various methods to characterize more complex fracture systems. Outcrop data characterization directly acquires near-surface fracture information through geological mapping or advanced remote sensing techniques like LiDAR and UAV photogrammetry. This approach represents the most direct and reliable method for studying fracture morphology, development processes, and statistical distribution patterns [12,13]. However, this method has limited applicability to deep rock masses, relying on indirect inferences from borehole, seismic, and other data, which are constrained by resolution and cost limitations [14]. To overcome these limitations, stochastic generation methods emerged. These methods construct random network models based on limited statistical data, assuming that fracture characteristics (e.g., location, length, orientation) follow certain probability distributions [15,16]. Building on this, fractal geometry theory was introduced to better characterize the heterogeneity, aggregation, and multi-scale characteristics of natural fracture systems [17,18]. For instance, Bour et al. [19] established a statistical model linking fracture density and length based on fractal dimension, while Lei et al. [20,21] utilized fractal theory for fracture network connectivity research and model upscaling. Nonetheless, traditional geometric and statistical methods still fall short in describing the connectivity between fractures and their regulatory mechanisms on the overall network behavior (e.g., connectivity).

In recent years, topological morphology characterization has provided a new perspective to address the above issues. Topology quantifies the complex connection structure of fracture networks by describing the spatial relationships among branches, nodes, and intersections. Sanderson and Nixon [22] pioneered this approach by decomposing two-dimensional fracture networks into sets of lines, nodes (classified as I-, Y-, and X-types), and branches. They established a set of quantitative indicators centered on node count, branch count, and connectivity. This method has been widely applied and developed. Morley and Nixon [23] revealed the intrinsic relationship between topological parameters and network maturity/connectivity through analysis of natural fault networks. Peacock et al. [24] and Duffy et al. [25] further emphasized topology’s effectiveness in describing network characteristics and understanding tectonic history. Santiago et al. [26] discovered classifiable topological patterns in rock samples and correlated them with tectonic activity history. Later, Sanderson et al. [27] combined graph theory with topological analysis to enhance the accuracy of fault and fracture system analysis. Numerous quantitative studies using topological methods [28,29] confirm that topological structure is an intrinsic key attribute of fracture networks and a crucial entry point for studying their formation mechanisms and evolution laws.

In revealing the mechanisms of hydraulic fracture propagation in fractured rock masses, laboratory experiments and numerical simulations are two indispensable research tools. In the laboratory, researchers apply hydraulic loading to rock specimens containing pre-existing fractures while using high-resolution observation techniques. These techniques include acoustic emission [30,31], CT scanning [7], and Digital Image Correlation (DIC) [5,32]. Using these methods, studies have systematically investigated the initiation, propagation, and coalescence of fractures, ranging from single and double fractures to more realistic scenarios like intersecting fractures and fracture networks [33,34,35]. For example, Zhao et al. [32] directly observed hydraulic fracture propagation in single-fracture granite specimens through visual experiments; Silva and Einstein [36] studied the physical processes of hydraulic fracturing; Fan et al. [37] and Liu et al. [38] experimentally explored hydraulic fracturing in orthogonal and random fracture networks, respectively. Regarding numerical simulation, the Discrete Element Method (DEM) such as Particle Flow Code (PFC) [39,40,41] and continuum-based methods like the Finite Element Method (FEM), Extended Finite Element Method (XFEM), and the emerging Phase-Field Method [42,43,44] are widely used to simulate fracture propagation.

Although existing methods have successfully reproduced various phenomena and offer advantages in revealing meso-mechanisms and multi-field coupling, most studies remain confined to single or simple multiple fractures. A significant gap exists in research that abstracts key topological configurations from complex networks to systematically investigate their influence on hydraulic fracture behavior. This includes their effects on competitive initiation, path deflection, and breakdown pressure. A prevalent but potentially incomplete view is that network complexity (e.g., more nodes and branches) invariably weakens rock mass strength. This view often overlooks the possibility that the topological configuration itself may regulate internal stress distribution, inducing nonlinear or even strengthening mechanical effects. Consequently, the mechanical mechanisms by which fracture topology controls hydraulic fracture propagation are still not well understood. Systematic research is especially scarce on how topological fractures affect hydraulic initiation locations, propagation paths, stress concentration, and multi-fracture interactions.

This study aims to reveal the mechanisms by which different types of topological fracture influence hydraulic crack initiation and propagation. Utilizing a comprehensive approach involving a visual hydraulic fracturing test system and improved discrete element numerical simulation methods, experimental and DEM numerical simulation studies were conducted on granite specimens containing 10 different topological fracture structures. The research focuses on elucidating the macroscopic control laws of topological nodes on crack initiation location, propagation path, breakdown pressure, and dynamic evolution behavior, and revealing the underlying mechanisms from the meso-mechanical perspective. The research results not only help bridge the gap in understanding complex fracture network behavior inherent in traditional single-fracture theories but also provide theoretical reference and technical support for the optimized design of shale gas fracturing, prevention and control of water inrush disasters in deep buried chambers, and efficient development of unconventional resources.

For clarity, the following terms are defined as used in this study: ‘Topology’ refers to the connectivity between nodes (endpoints, intersections) and branches within a fracture network. ‘Topological structure’ denotes the specific spatial arrangement of these nodes and branches. ‘Fracture network’ refers to the system composed of the fracture entities themselves.

## 2. Materials and Methods

### 2.1. Characterization of Fracture Topology and Specimen Preparation

**Figure 1:**
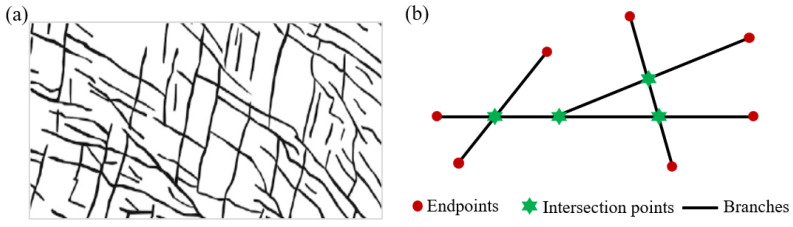
(**a**) Schematic diagram of natural fracture network; (**b**) Simplified topological nodes.

Under the influence of tectonic movements, unloading, and weathering, various natural fractures of different scales and quantities form inside and on the surface of rock masses. These fractures collectively constitute the internal fracture network structure of the rock mass (as shown in Figure 1a). Fracture intersection nodes and endpoints often exhibit significant stress singularity and hydraulic path mutations [1,21]. To quantitatively study the influence of topological structure, this paper first proposes a simplified characterization method based on topological nodes, abstracting complex natural fracture networks into basic topological units such as I-shape, T-shape, X-shape, and their combinations. This establishes a framework for systematic and comparable experimental research on the hydraulic behavior of fracture networks.

For complex fracture networks, topological simplification is performed, assuming all fractures can be approximated by combinations of line segments, and considering the fracture network to consist of fracture nodes and branches. As shown in Figure 1b, nodes can be divided into intersection nodes and endpoints. The fractures between nodes are called branches. Endpoints are connected to only one branch, while intersection nodes are connected to at least two branches in different directions. Mathematically, the nodes of the fracture network are defined as a series of discrete points in the study domain Ω:

(1) $$𝒩=\left\{n_{1},n_{2},\cdots ,n_{M}\right\}$$

where M is the total number of nodes. Each node $n_{i}\in N$ has a unique position coordinate $p_{i}=\left(x_{i},y_{i}\right)$ or $p_{i}=\left(x_{i},y_{i},z_{i}\right)$ in space. Based on the number of topologically connected branches, nodes can be classified as endpoints or intersection points. A node type function can be defined:

(2) $$\tau (n_{i})=\left\{\begin{array}{cc} \;Endpoints & \;\mathrm{if}\;\deg(n_{i})=1\\ Intersection\;points & \;\mathrm{if}\;\deg(n_{i})\geq 2 \end{array}\right.$$

where $\deg(n_{i})$ represents the number of branches directly connected to node $n_{i}$. Physically, endpoints are located at the ends of fracture paths, representing the start/end points of fractures or the termini of unconnected fractures; endpoints are often points of stress concentration, potential initiation or termination locations for crack propagation paths. Intersection points represent the points where fractures intersect; branches of different directions connect here, where fluid converges, diverges, or mixes, making them key hubs for fluid flow within the fracture network.

The branches of the fracture network are the basic units forming the fracture paths by connecting nodes, represented by a collection of line segments, defined as:

(3) $$ℬ=\left\{b_{1},b_{2},\cdots ,b_{M}\right\}$$

where K is the total number of branches. Each branch $b_{k}$ is uniquely determined by the two nodes it connects, denoted as $b_{k}=\left\{n_{sk},n_{ek}\right\}$, where $n_{sk},n_{ek}\in 𝒩$ and $n_{sk}\neq n_{ek}$. Geometrically, $b_{k}$ is the line segment connecting nodes $n_{sk}$ and $n_{ek}$, which can be represented in space by the linear equation:

(4) $$L_{k}=\left\{x\in R^{d}|x=(1-t)P_{sk}+tP_{ek},0\leq t\leq 1\right\}$$

The attributes of each branch of the fracture network can be calculated based on its nodes. Its length is determined by the Euclidean length of the branch:

(5) $$L_{k}={\left\|P_{ek}-P_{sk}\right\|}_{2}$$

### 2.2. Preparation of Specimens with Simplified Topological Structures

**Figure 2:**
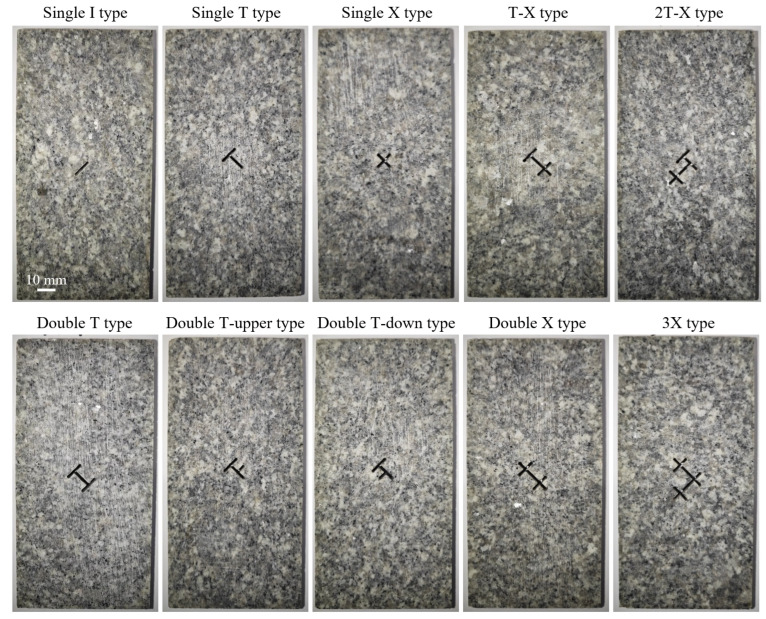
Designs of the ten pre-existing fracture specimens with different topological structures.

Based on the characterization method in the previous section, and to further simplify and conduct experimental research, three basic node types can be derived based on network structure characteristics: I-shaped nodes (isolated fractures), T-shaped nodes (triple junctions), and X-shaped nodes (cross intersections). Through combination and extension, 10 topological fracture morphologies were systematically designed for specimen preparation and experimental study (Figure 2), including simple nodes (single I, single T, single X), homogeneous combination nodes (double T, double T upper, double T lower, double X, 3X), and mixed nodes (T-X, 2T-X), achieving coverage from simple to complex topological structures. It should be noted that the selection of these ten configurations is based on topological principles rather than the direct simulation of any specific natural pattern. The I, T, and X nodes represent the elementary topological units in 2D fracture networks. The combined configurations form a logical sequence that allows us to isolate and investigate the effects of two key factors: topological complexity and spatial configuration. While these simplified geometries encompass the common connection patterns (e.g., intersections, branches) observed in natural fracture networks, the primary aim of this study is to reveal the fundamental control mechanisms of topology on hydraulic fracturing behavior, providing a theoretical basis for understanding more complex, realistic scenarios.

The experiments used Befast granite from Pingyi, China, with mechanical parameters [32] as shown in Table 1. According to the International Society for Rock Mechanics standards, the specimens were processed into rectangular blocks with a width of 75 mm, height of 150 mm, and thickness of 20 mm. All pre-existing fractures were prepared using high-precision water-jet cutting technology to ensure accurate realization of complex geometric shapes. Referring to similar experimental studies on specimens with pre-existing fractures and considering boundary effects and water-jet machining accuracy, the main pre-existing fracture length 2a was determined to be 11 mm, the single branch length 5.5 mm, and the width 1 mm. The number of endpoints, intersection nodes, branches, and the normalized total fracture length for each type of topological structure specimen are shown in Table 2. The normalized total fracture length is defined as:

(6) $$\tilde{L}=\frac{\sum L_{k}}{a}$$

For DIC analysis of the hydraulic fracture propagation process, a high-contrast matte black and white speckle pattern was sprayed onto the observation surface of the specimens. The black and white speckles further enhanced the image quality for DIC processing based on the granite’s inherent color; the spray paint used was TAMIYA TS-27 White and TS-6 Black, which remains stable under the high water pressure of the test environment.

### 2.3. Visual Hydraulic Fracturing Test System

**Figure 3:**
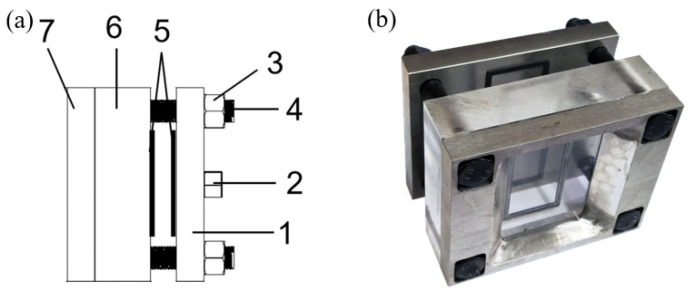
(**a**) Sealing device structural composition: 1—stainless steel back plate, 2—water injection hole, 3—high-strength nut, 4—high-strength bolt, 5—rectangular fluoroelastomer seal ring and groove, 6—thick acrylic observation window, 7—stainless steel front plate; (**b**) Physical image of the sealing device.

Revealing the influence mechanism of topological fracture morphology on hydraulic crack initiation and propagation requires obtaining the evolution process from damage accumulation to crack propagation. This study achieved direct observation of the fracturing process based on an advanced test system. The experiments relied on a visual hydraulic fracturing device developed by the team [32,45], which successfully resolves the contradiction between “high water pressure sealing” and “visualization”. Improved based on the team’s previous research, the device, as shown in Figure 3, uses a 40 mm thick high-strength polycarbonate plate as the observation window. Sealing is achieved using a rectangular-section fluoroelastomer seal ring and matching seal groove. After tightening with high-strength bolts, stable sealing is maintained under 15 MPa water pressure for a relatively long duration.

**Figure 4:**
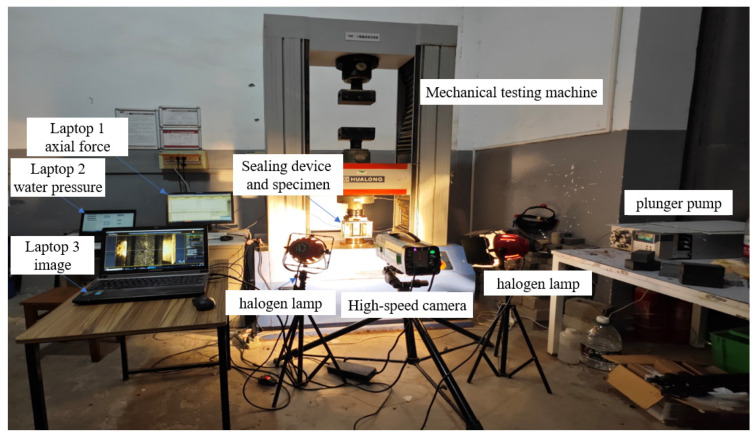
Schematic diagram of the integrated visual hydraulic fracturing test system.

The test system is shown in Figure 4 and consists of three parts: (1) Axial loading system: a WDW-600 universal testing machine produced by Shanghai Hualong Test Instrument Co., Ltd. (Shanghai, China), providing constant axial pressure; (2) Fluid pressure application and monitoring system: an EX-1600 plunger pump from US-based Yima Technology enables constant flow injection; an MD-G pressure transducer from Shanghai Mingkong Sensing Technology Co., Ltd. (Shanghai, China). and a data acquisition card record water pressure in real-time at 60 Hz; (3) Optical recording system: an i-speed 716 high-speed camera (maximum resolution 1920 × 1080 pixels, minimum shutter speed 1 μs, maximum frame rate 500,000 fps, featuring loop, ROC, and BROC trigger modes) fitted with a Nikon 100 mm lens via a C-mount adapter records the crack propagation process throughout, with two halogen lamps for illumination. The three test subsystems are independently controlled by three computers, responsible for axial force servo control, water pressure acquisition, and image acquisition, respectively. Data acquisition from each computer is synchronized by aligning time.

### 2.4. Test Procedure and DIC Analysis

Building upon previous uniaxial compression tests conducted on five types of fractured rock mass specimens with topological characteristics (I-type, T-type, double T-type, T-X-type, and double X-type) [46], this study performed hydraulic fracturing tests on all 10 types of topological fracture specimens. The specific procedure is as follows: After installing the specimen into the sealing device and placing it on the testing machine loading platform, preload the specimen to 50 N. Then, adjust the shooting parameters of the high-speed camera to ensure the captured images are clearest, and fine-tune the specimen position so the pre-existing fracture is centered in the frame. Subsequently, apply load at a rate of 0.2 kN/s until the load reaches 15 kN (i.e., 10 MPa) and then maintain constant axial pressure. Next, take an initial photo as a reference for DIC calculation, then start the water pressure monitoring software and high-speed camera recording. Inject water at a constant flow rate of 5 mL/min until hydraulic fracturing is induced, then stop data acquisition and save the data. The high-speed camera acquisition resolution is 1280 × 1536, frame rate 300 fps, continuously acquiring for about 120 s before overwriting. Post-trigger control stops acquisition after fracturing failure, ensuring the fracturing process is recorded. To reduce the impact of random errors, each test group was repeated 3 times, labeled A, B, and C.

After the tests, representative images from a period before crack propagation were selected, and the DIC method was used to analyze the damage evolution and macroscopic crack propagation process. The analysis employed an improved software version [47] based on open-source Ncorr [48]. This software utilizes adaptive subset partitioning to accurately capture discontinuous deformation fields near cracks. The correlation criterion is the zero-mean normalized sum of squared differences (ZNSSD), where the correlation coefficient typically ranges from 0 to 4, with smaller values indicating higher similarity. The improved algorithm automatically performs subset segmentation and recalculates for points where the correlation coefficient exceeds a set threshold, significantly improving displacement calculation accuracy near crack tips. Detailed descriptions of these improvements can be found in Tian et al. [47]. For the DIC analysis in this study, the key parameters were set as follows: a subset size of 15 pixels, a step size of 0 pixels (meaning calculations were performed at all pixel points), a correlation coefficient cutoff value of 0.3, and a strain calculation radius of 10 computation points for fitting the strain field from the displacement data.

For ease of image selection, the moment when the macroscopic crack becomes visible to the naked eye is defined as time 0. The frame rate is used to count backward, and the image time is recorded as the time interval from the macroscopic crack generation. Based on the DIC analysis results, the influence mechanism of topological fractures on hydraulic crack propagation was primarily analyzed through the evolution of the major principal strain field and the crack opening process.

## 3. Macroscopic Influence Laws of Topological Structure Fractures on Hydraulic Crack Propagation

To directly reveal the control effect of topological structure on crack propagation under hydraulic driving, this section, based on visual hydraulic fracturing tests combined with DIC full-field strain analysis, systematically elaborates the macroscopic influence laws of topological nodes from four dimensions: crack initiation location, propagation path, breakdown pressure, and dynamic evolution behavior.

### 3.1. Influence of Topological Structure on Hydraulic Crack Initiation Location

Based on visual observation and DIC strain field analysis, the hydraulic cracks in all topological fracture specimens exhibited clear initiation and path selection rules. The experimental results indicate that regardless of the complexity of the topological structure, the hydraulic crack initiation location always occurs near an endpoint and strictly follows the “proximal-to-loading-end priority” rule. That is, the branch fracture tip closest to the axial loading end becomes the advantageous location where strain energy is most easily released and cracks are most prone to initiate.

**Figure 5:**
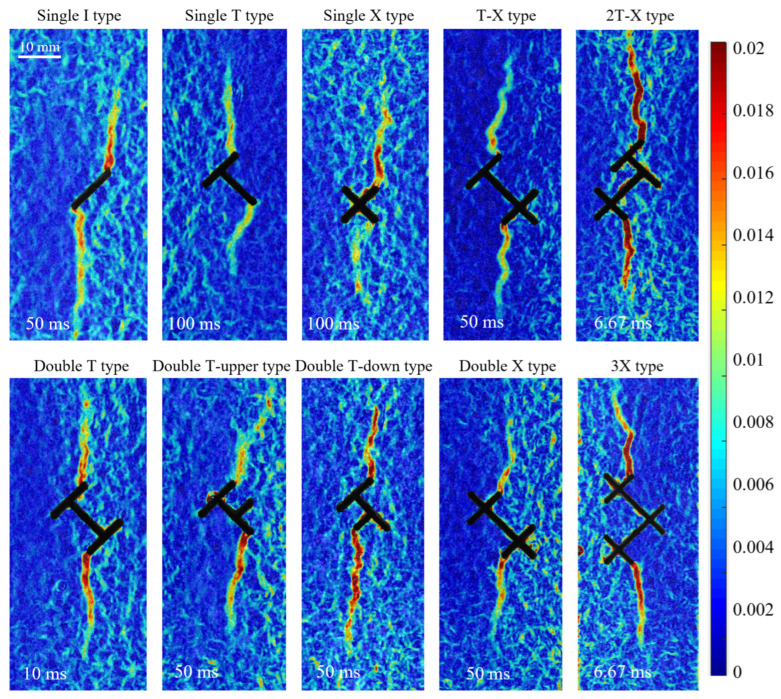
Major principal strain field near typical topological nodes before macroscopic crack propagation (Group A topological nodes nearby). The time in the figure indicates the time interval from that moment to the time of macroscopic crack propagation.

As shown in Figure 5, for the I-shaped fracture, strain localization zones (SLZs) initiate simultaneously at both the upper and lower ends of the pre-existing fracture; for the X-shaped fracture, SLZs initiate at two symmetric tips. For all other fractures, cracks initiate from the branch fracture tip closest to the loading end. It is worth noting that except for the symmetric I-type and X-type nodes, for T-type topological node fractures, double T-shaped topological node fractures, and double T upper-shaped topological node fracture specimens, the optimal location at the upper and lower ends closest to the tip is unique. However, for double T lower and other mixed nodes combined with X (T-X shape, 2T-X shape), there are two equidistant tips at the lower part; for the 2X shape and 3X shape topological nodes, there are two equidistant tips at both the upper and lower parts. The experimental results consistently show that when there are two equidistant tips, the hydraulic crack always initiates at the tip closer to the center of the specimen. The specimen is only subjected to vertical uniaxial load during the test. We believe that selecting the tip closer to the center is because it is farther from the zero-stress boundary of the specimen’s side surface.

The highly consistent experimental results regarding the crack initiation location indicates that the relative distance between the topological node and the loading boundary is the dominant factor determining the hydraulic crack initiation location. Under constant far-field stress, the spatial orientation of the topological node essentially affects the stress field at the tips of each pre-existing crack. Therefore, based on the experimental results, it can be reasonably inferred that the influence of the stress field distribution at the tips of fractures with specific topological structures on the hydraulic crack initiation location surpasses the influence of the node’s own local geometric configuration.

### 3.2. Influence of Topological Structure on Hydraulic Crack Propagation Path

**Figure 6:**
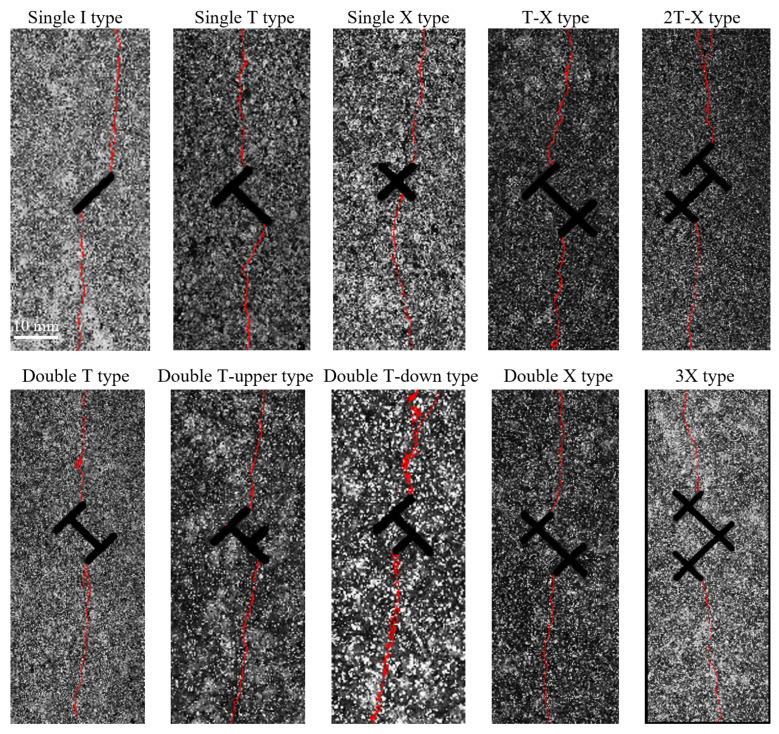
Hydraulic crack paths (marked in red color) for ten pre-existing fracture specimens with different topological structures (near the topological nodes).

Based on DIC-identified macroscopic crack locations, the hydraulic crack propagation paths can be analyzed, as shown in Figure 6. Overall, the main propagation direction of hydraulic cracks in all specimens ultimately tends to align with the axial maximum principal stress direction. Regardless of whether the hydraulic crack deflects or not after initiation, the final macroscopic crack path always extends towards the specimen loading end. However, during the initial stage of initiation, the crack path can exhibit significant deflection influenced by the local topological geometry.

Crack paths can be morphologically classified into Type I tensile crack and Type Ib tensile crack (referring to Zhao et al. [32]), where Type I tensile crack exhibits wing crack characteristics, while Type Ib tensile crack shows no significant deflection at the initiation location. For example, the hydraulic crack in the Single I type extends almost straight vertically towards the specimen loading end. Hydraulic cracks formed by other topological nodes may have a large angle between the crack surface and the pre-existing fracture at initiation, exhibiting obvious “turning” behavior, later adjusting to become parallel to the principal stress direction, resembling the characteristics of Type I tensile wing cracks. Additionally, local path bending was observed during the propagation of some hydraulic cracks; local tortuosity does not affect the overall crack path. We believe that local tortuosity is related to the distribution of mineral grains in the granite specimen.

Due to experimental limitations, hydraulic fracturing tests were only conducted under uniaxial stress. Judging from the overall results, the final macroscopic hydraulic crack paths also all start from near the endpoints and extend towards the specimen ends. The direction of the maximum principal stress has a stronger guiding effect on the final crack path. This insight was also confirmed in subsequent numerical simulation studies.

### 3.3. Dual Influence of Topological Structure on Breakdown Pressure: Weakening Effect and Configuration Strengthening

**Figure 7:**
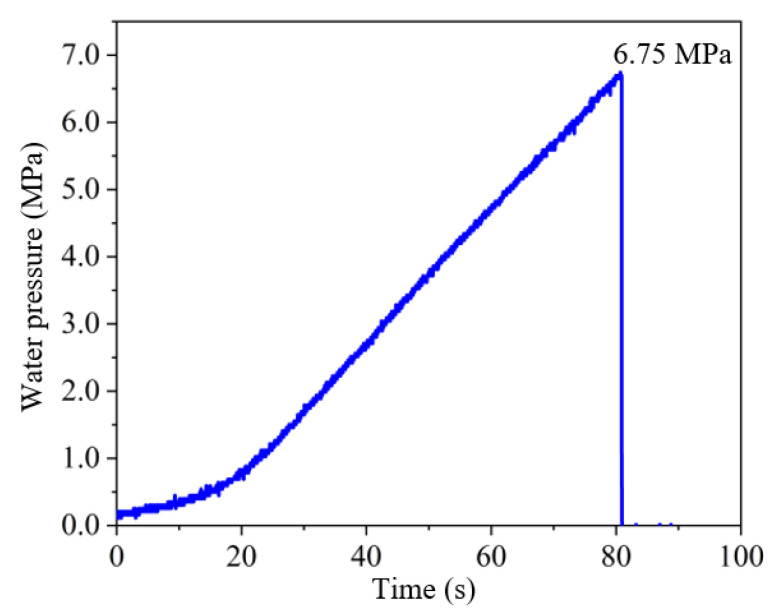
Fluid injection curve for Single T type, Group A specimen.

The fluid injection curves for topological fractures share common typical characteristics. As shown in the injection curve for the Single T type, Group A specimen in Figure 7, the water pressure rises nonlinearly initially as the fluid fills the fracture and compresses the specimen and sealing mold. Subsequently, due to the low compressibility of the fluid, the water pressure increases almost linearly until it reaches a peak, inducing hydraulic fracturing in the specimen. Because macroscopic hydraulic crack coalescence leads to sealing failure, the water pressure drops to zero within a very short time after the peak.

The breakdown pressure, particularly the breakdown pressure, is a key indicator of the rock mass’s resistance to fracturing. To accurately quantify the influence of topological structure, this section analyzes the weakening effect of topological node type and quantity on the breakdown pressure. The breakdown pressure test results for the ten types of topological fracture specimens are shown in Table 3. Combined with the topological quantitative parameters of each specimen type (Table 2), the weakening effect of topological nodes on the breakdown pressure is systematically analyzed.

**Figure 8:**
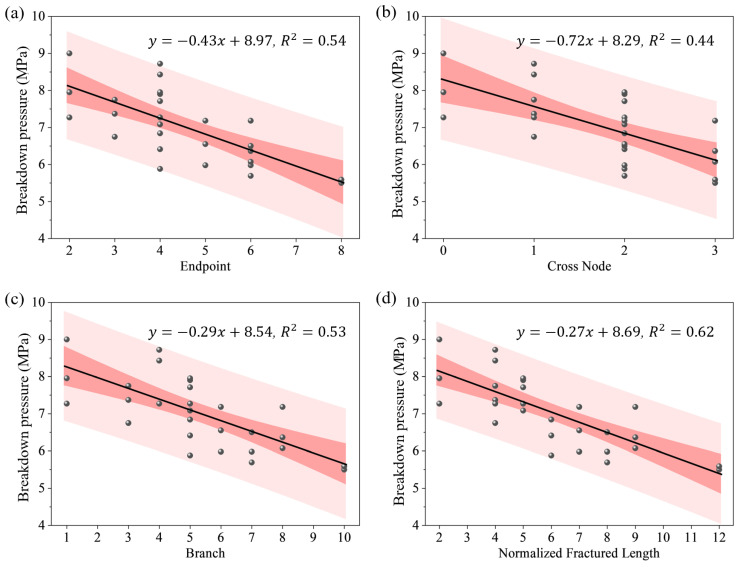
Correlation analysis between breakdown pressure and topological quantitative parameters: (**a**) Endpoints, (**b**) Intersection nodes, (**c**) Number of branches, (**d**) Normalized total fracture length. The solid line in each graph represents the linear fit, with its equation and coefficient of determination (R^2^) provided. The slopes of all regression lines are statistically significant (*p* < 0.001). The inner pink band and the outer light red band represent the 95% confidence band and the 95% prediction band, respectively.

To reveal the systematic influence of topological structure, we conducted a correlation analysis between the breakdown pressure and the topological parameters. As shown in Figure 8, despite a certain degree of data scatter, the fitted linear trends, along with their confidence and prediction bands, consistently indicate a significant negative correlation between the breakdown pressure and all four topological parameters: the number of endpoints, intersection nodes, branches, and the normalized total fracture length. The linear regression equations are displayed in each subplot of Figure 8. The coefficients of determination (R^2^) range from 0.44 to 0.62, confirming a moderate to strong correlation. Analysis of variance (ANOVA) confirms that all fitted slopes are statistically highly significant (*p* < 0.001 for all), robustly supporting the declining trend. We interpret this as evidence that an increase in topological nodes and fracture length effectively raises the initial damage density within the rock mass. Consequently, the specimen behaves as if it possesses greater pre-existing damage. Furthermore, a higher number of endpoints provides more stress concentration points and potential sites for crack initiation. Therefore, the experimental data demonstrate that increasing the complexity of the topological structure significantly reduces the breakdown pressure required for hydraulic fracturing.

The relatively narrow 95% confidence bands (inner pink bands) in Figure 8 indicate a high precision in the estimated regression slope describing the reduction in breakdown pressure with increasing topological parameters. However, the considerably wider 95% prediction bands (outer light red bands) imply that accurate prediction of the breakdown pressure for a new specimen with specific topological parameters is challenging using linear regression alone. This quantitative feature, highlighted by the wide prediction bands, statistically underscores the critical role of fracture topological ‘configuration’ in controlling the breakdown pressure. This observation aligns well with the experimental results: specimens of the same type, or those with identical topological parameters but different spatial configurations (e.g., ‘Double T-upper’ vs. ‘Double T-lower’), exhibited variations in their breakdown pressures. Furthermore, specimens with more complex topological parameters sometimes demonstrated higher measured pressures, such as the X-shaped type showing a higher breakdown pressure than the T-shaped type, and the T-X type showing a higher breakdown pressure than the Double T type.

Another point worth discussing is that among the single node types, the average breakdown pressure for the single X-shaped fracture (8.14 MPa) is higher than that for the single T-shaped fracture (7.29 MPa), which seems contradictory to the overall macroscopic trend of lower breakdown pressure with more nodes. We believe this phenomenon can be explained by the mechanical mechanisms of the two fracture structures: Under axial load, the T-shaped topological structure has more significant stress concentration at its upper and lower two tips, while the cross structure of the X-shaped node causes the stress concentration due to external loading to be dispersed among its four endpoints. The difference in their mechanical mechanisms causes the T-shaped topological structure to undergo hydraulic fracturing at a lower water pressure compared to the X-shaped fracture.

In summary, based on quantitative experimental data, this study confirms that the hydraulic fracturing characteristics of fracture networks are significantly influenced by their topological structure overall. The spatial distribution form of the fracture structure and the stress differences caused by the in situ stress field also affect the breakdown pressure.

### 3.4. Dynamic Evolution Behavior of Hydraulic Cracks

**Figure 9:**
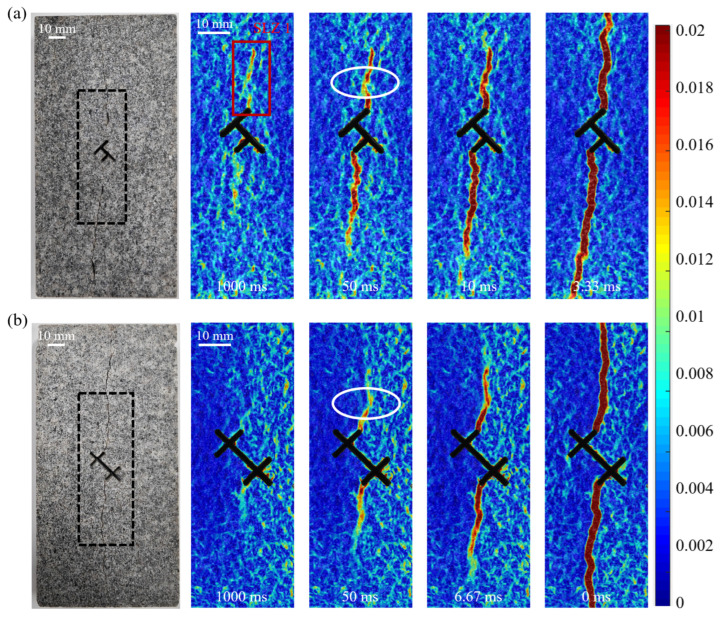
Evolution process of the major principal strain field near pre-existing fractures during hydraulic fracturing (Group A): (**a**) Double T-down type; (**b**) Double X-type. The red box marks the initial strain localization zone (SLZ 1). The white dashed ellipse highlights the discontinuous strain localization zone indicative of intermittent crack propagation.

To deeply understand the dynamic process of crack propagation, we used DIC technology to analyze the global strain field and quantified the dynamic opening behavior of cracks through the displacement jump at characteristic points. DIC analysis revealed microscopic precursors before hydraulic crack initiation. Before the macroscopic crack was visible, strain localization zones (SLZs) had already formed in areas of high strain rate. As shown in Figure 9, taking Group A Double T-down type as an example, at 1 s before fracturing, patchily distributed strain concentration zones SLZ 1 (red box) can be observed near the pre-existing crack tip. Subsequently, the damage degree of the specimen rapidly increased, forming a through-going strain localization zone before the macroscopic crack appeared, and the strain localization zone completely coincided with the final hydraulic crack location. During the crack propagation process, we observed in some specimens that the crack did not propagate continuously. As shown in the area within the white elliptical box in the figure, the strain localization zone here is clearly discontinuous, indicating that this area lagged behind adjacent regions during the crack opening process. This intermittent propagation phenomenon updates the traditional view that hydraulic cracks propagate continuously. We believe that the occurrence of intermittent propagation is related to the heterogeneity of the granite.

**Figure 10:**
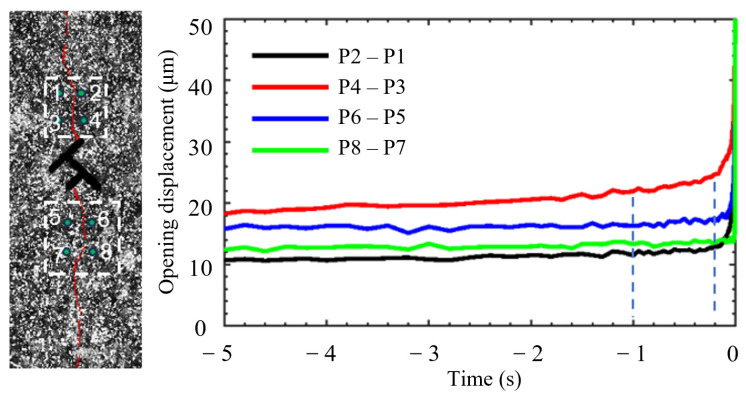
Analysis of the crack opening process for Double T-down type, Group A specimen. The numbers in the dashed box in the left figure indicate the positions of the query points taken for analyzing the characteristic displacement. The curve in the right figure shows the relative horizontal displacement difference for four pairs of query points. The horizontal coordinate time uses the failure moment as the origin, i.e., negative values represent before failure. At time 0, the macroscopic crack coalesces, and the relative displacement is maximum.

Regarding the propagation evolution process of hydraulic cracks, by monitoring the horizontal displacement difference between characteristic points on both sides of the crack, the crack opening process can be precisely quantified. The displacement jump curves of all specimens exhibit similar characteristics. Taking the opening process shown in Figure 10 as an example, about 1–2 s before the breakdown pressure, the displacement difference begins to increase steadily, marking the stable propagation of the crack; about 0.2–0.5 s before the breakdown pressure, the displacement difference increases sharply, marking the crack entering the unstable rapid propagation stage until the specimen completely fails. The relative displacement change in Figure 10 confirms that local damage occurred before the macroscopic crack opened, and the damage degree intensified nonlinearly. From the evolution process, no significant influence of topological structure on the opening process of a single hydraulic crack was observed. The crack opening process is consistent with the cohesive zone model.

## 4. Hydraulic Crack Propagation Mechanisms Based on DEM

### 4.1. Hydromechanically Coupled Discrete Element Model and Numerical Simulation Scheme

#### 4.1.1. Model Establishment and Parameter Calibration

To reveal the hydraulic propagation mechanisms of topological fractures from the meso-scale, this study used the Flat-Joint Model (FJM) in PFC2D to construct a hydromechanically coupled numerical model. The core of this model lies in simulating fluid flow and pressure transmission within an assembly of particles. The fluid is assumed to exist in “flow domains” constituted by lines connecting particle centers. Adjacent domains are connected by “channels”. The opening/closing state of the channels is dynamically linked to the fracture of contacts between particles, thereby achieving full coupling between crack propagation and fluid flow.

**Figure 11:**
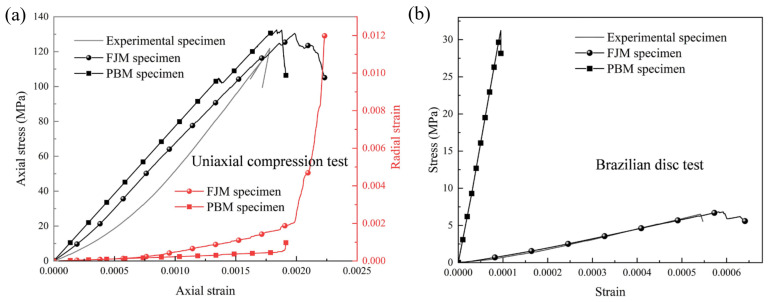
Comparison of numerical simulation and experimental results obtained from stress–strain curves [41]: (**a**) uniaxial compression and (**b**) Brazilian disk tests.

The model geometry is consistent with the laboratory tests. Particles were generated using a recursive algorithm, and boundary constraints were applied. A stable initial model was obtained after self-equilibrium calculation. Based on the macro-mechanical parameters of granite measured in the laboratory (Table 1), the meso-parameters of the FJM were systematically calibrated using a trial-and-error method. The calibration outcome is presented in Figure 11, which compares the stress–strain curves obtained from the numerical simulation and laboratory experiments under uniaxial compression and Brazilian disk test conditions. The results demonstrate that the mechanical properties of the calibrated FJM specimen are in good agreement with those from the experiments. Compared to the Parallel Bond Model (PBM), which typically exhibits an unrealistically high tensile strength, the FJM adopted in this study can better reproduce the actual compressive-to-tensile strength ratio of the rock. The final determined meso-mechanical and hydraulic parameters are listed in Table 4. Detailed information regarding the model algorithm and parameter calibration procedure can be found in the team’s previously published paper [41].

#### 4.1.2. Numerical Simulation Scheme and Verification

The fracture topological structure morphologies used in the numerical tests are consistent with the visual tests, i.e., identical pre-existing crack combinations as in Figure 2. The test setting maintained a vertical pressure of 10 MPa, with boundary conditions consistent with the experiments.

**Figure 12:**
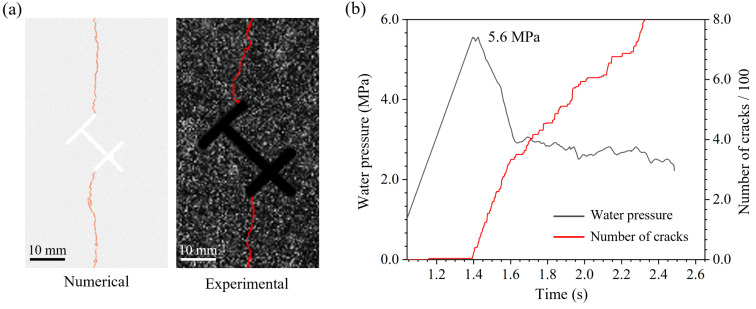
Numerical simulation well reproduces the experimental results: (**a**) Crack propagation path comparison; (**b**) Water pressure curve from numerical simulation.

**Figure 13:**
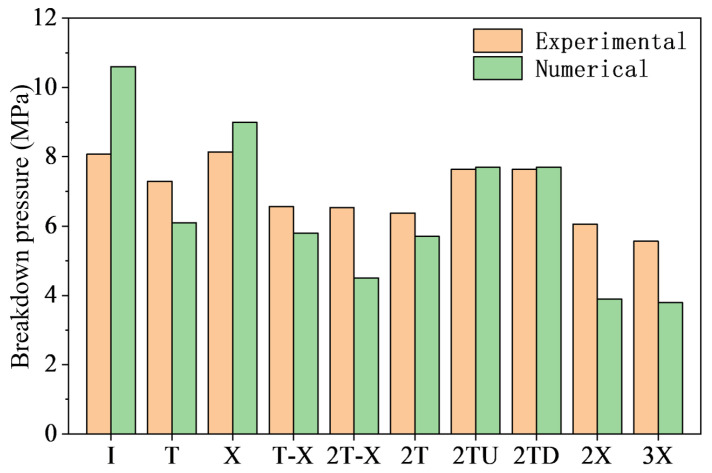
Comparison of average breakdown pressure between experimental and numerical simulation results.

To verify the model’s reliability, we compared the numerical simulation results with the laboratory tests. The results show that the final crack propagation morphology obtained from the numerical simulation is highly consistent with the experimental results. Taking the T-X type specimen shown in Figure 12a as an example, the numerical simulation reproduced the entire process observed in the experiment: crack initiation from the upper and lower node branch tips, propagation path deflection, and final vertical coalescence. Even local deflection characteristics were accurately reproduced. Regarding the water pressure curve, since macroscopic crack propagation leads to sealing failure in the experiment, the breakdown pressure is the most valuable quantitative comparison indicator. As shown in Figure 12b, the simulated breakdown pressure for the T-X type specimen is 5.6 MPa, close to the experimental average of 6.5 MPa, with the gap within a reasonable error range. Figure 13 shows the comparison of the average breakdown pressure between the experimental and numerical results. The relative magnitudes and trends for each specimen remain consistent. The comparison of simulation results fully verifies that this hydromechanically coupled discrete element model can effectively simulate the hydraulic fracturing process of rocks containing topological fractures.

### 4.2. Meso-Mechanism of Topological Structure Influencing Hydraulic Crack Propagation

**Figure 14:**
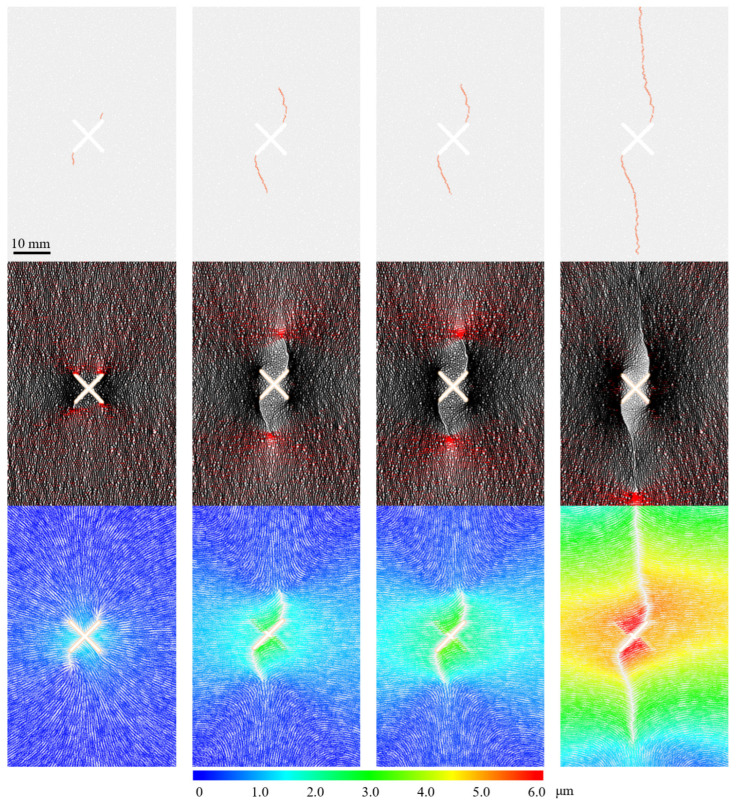
Macroscopic crack path, force chain distribution, and displacement field distribution at different crack propagation stages for the X-shaped node fracture. The macroscopic crack path is visualized by broken contacts, colored in red. In the force chain diagrams, red indicates tensile contact between particles, and black indicates compressive contact.

Based on discrete element numerical simulation, the meso-mechanisms of hydraulic crack propagation can be analyzed in detail. The most core finding of the numerical simulation is that it reveals how the topological structure fundamentally determines the hydraulic crack initiation behavior by regulating the distribution pattern of the meso-stress field. Taking the X-shaped node fracture as an example, Figure 14 shows the meso-evolution sequence during its hydraulic fracturing process under uniaxial stress, displaying the macroscopic crack path, force chains, and displacement vectors at different crack propagation stages. During the crack initiation stage, obvious stress concentration can be observed at all four crack tips from the force chains, but the macroscopic hydraulic crack initiates from one tip each near the upper and lower loading ends and continuously extends. As the crack propagates, the concentration area of tensile force chains (red) gradually extends towards the advancing macroscopic crack tip. From the displacement field, it can be seen that the displacement difference across the crack gradually increases, i.e., the hydraulic crack opens progressively, consistent with the trend observed in experiments based on DIC. As the crack tips continuously extend towards the specimen ends, the specimen eventually fractures. The displacement vector diagram indicates that the entire failure process is dominated by tensile failure, with only local shear vortices observed near the tips during the initial stage of crack initiation.

**Figure 15:**
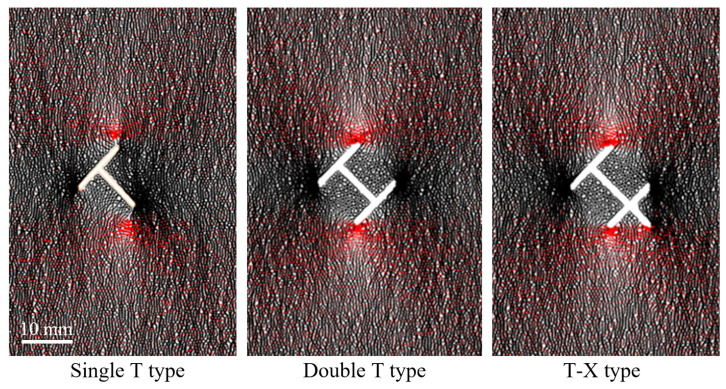
Force chain diagrams for representative specimens at breakdown pressure. Red indicates tensile contact between particles, and black indicates compressive contact.

It is noteworthy that before hydraulic crack initiation, the force chains clearly show that the tensile stress concentration in the X-type specimen is dispersed to the tips of the four branch fractures, forming a “multipolar” stress distribution. In contrast, the T-type specimen shows stress concentration at one tip each at the upper and lower parts at breakdown pressure (as shown in Figure 15). The X-type topological fracture has both a higher number of nodes and branches than the T-type fracture. According to the more general rule, the breakdown pressure for the X-type should be lower than for the T-type. However, both in experiments and numerical simulations, the X-type topological fracture was observed to have a higher breakdown pressure. Based on the differences in stress distribution observed in the numerical simulation, we believe that the simultaneous stress concentration at multiple endpoints in the X-type fracture reduces the strain energy density near individual tips, thus requiring a higher overall water pressure to trigger initiation. This perfectly explains the “anomalous” phenomenon of breakdown pressure observed in experiments and numerical simulations from a mechanical perspective.

**Figure 16:**
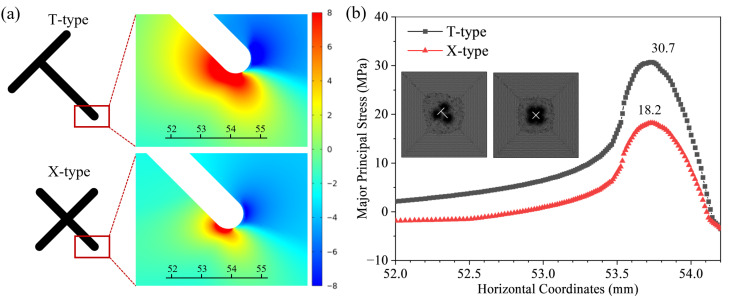
(**a**) Distribution of maximum principal stress near the crack tips for the X-type and T-type configurations from supplementary elastic FEM analysis (axial load = 10 MPa, internal fluid pressure = 5 MPa). (**b**) Maximum principal stress values along a path adjacent to the pre-exsisting fracture wall, highlighting the significant difference in peak stress concentration between the two topological types.

To provide a more quantitative validation of this “dispersed stress concentration” mechanism, supplementary linear elastic finite element analysis was conducted. The model simulated the pre-initiation state under an axial load of 10 MPa and a fluid pressure of 5 MPa uniformly applied inside the pre-existing fractures. The distribution of the maximum principal stress near the crack tips is shown in Figure 16a. Under identical external loading, the T-type configuration exhibits a much more intense stress concentration at its lower tip compared to any tip in the X-type configuration. The maximum principal stress values extracted along a path adjacent to the fracture wall (Figure 16b) reveal that the peak tensile stress at the critical tip of the T-type specimen reaches 30.7 MPa, which is substantially higher than the 18.2 MPa observed at the tips of the X-type specimen. This elastic mechanics analysis quantitatively confirms that, for the same boundary conditions, the X-shaped node induces a lower level of stress concentration at its individual tips due to stress dispersion, thereby requiring a higher fluid pressure to initiate hydraulic fracturing. This finding provides direct numerical support for the counterintuitive higher breakdown pressure of the X-type node.

In more complex topological combinations, similar phenomena also exist in Double T type and T-X type. As shown in Table 2, T-X type has one more endpoint, one more branch, and a longer normalized fracture length compared to Double T type. That is, the T-X type specimen has greater initial damage and is thus expected to have a lower breakdown pressure. In fact, as shown in Table 3, the breakdown pressures for the three T-X type specimens in the experiment are 6.55, 5.98, and 7.18 MPa, average 6.57 MPa, while for the three Double T type specimens they are 6.41, 5.88, and 6.84 MPa, average 6.38 MPa. The breakdown pressure for T-X type is higher than for Double T type, and this difference is not caused by discrete errors of individual specimens. In the numerical simulation results, the breakdown pressure for T-X type (5.8 MPa) is also slightly higher than for Double T type (5.7 MPa), consistent with the experimental results. The force chain diagram in Figure 14 can very intuitively explain this “anomalous” phenomenon. The stress concentration at the two lower tips in the T-X type requires a higher water pressure to drive crack propagation. The strengthening effect due to dispersed stress concentration outweighs the weakening effect of greater initial damage. Therefore, after the two effects cancel each other out, the breakdown pressure of T-X type remains slightly higher than that of Double T type.

**Figure 17:**
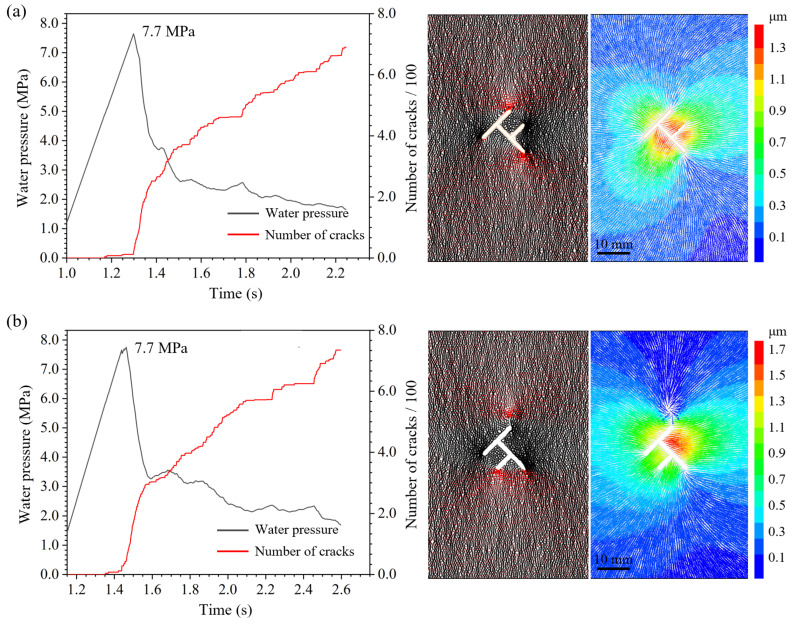
Water pressure curve and force chain diagram and displacement diagram at breakdown pressure: (**a**) Double T-upper type; (**b**) Double T-down type.

A particularly insightful aspect of the experimental design in this study involves the comparison between the Double T-upper and Double T-down topological structures. When characterized using the topological node method illustrated in Figure 1, these two configurations possess identical topological parameters: the same number of endpoints, intersection nodes, branches, and normalized fracture length. The sole geometric distinction is that the Double T-down type features two initial crack tips in its lower section that are equidistant from the specimen boundary. Based on the previously discussed meso-mechanical mechanism, one would logically predict the Double T-down type to require a higher breakdown pressure due to the dispersion of stress concentration between its two lower tips. However, the experimental results revealed a remarkable finding: the average breakdown pressure for the Double T-down type was identical to that of the Double T-upper type (7.64 MPa). The authors meticulously verified the experimental data and confirmed the coincidence. This identical peak pressure was also reproduced in the numerical simulations, where both the Double T-upper and Double T-down types yielded the same value, as shown in Figure 17.

Detailed analysis of the force chains and displacement fields from the simulation provided the explanation: in both configurations, hydraulic cracks initiated preferentially from the single upper tip. Consequently, the shared stress concentration at the two lower tips in the Double T-down type did not govern the failure process. The breakdown pressure for both specimens was ultimately dictated by the strength of the first-to-initiate upper tip. This result further underscores that the topological structure governs hydraulic crack initiation behavior by modulating the internal stress field, and that the critical initiation site—not merely the parameter counts—determines the macroscopic fracturing response.

In summary, based on simplified topological fractures, integrating experimental results and numerical simulation analysis from the meso-mechanical perspective, we conclude that the topological structure of fracture networks influences crack initiation and propagation behavior by affecting the stress distribution within the rock mass. More complex geometric combinations, i.e., those with more nodes, branches, and length, are equivalent to greater initial damage, weakening the rock mass strength and making hydraulic fracturing easier. Differences in stress concentration at the tips caused by different fracture topological structures also affect the hydraulic fracturing of fractured rock masses.

## 5. Discussion

### 5.1. Principal Findings and Implications

This study, through visual hydraulic fracturing tests and discrete element numerical simulation, systematically revealed the macroscopic laws and meso-mechanical mechanisms of topological structure on hydraulic crack propagation. The simplified topological structure method based on nodes (endpoints, intersection points) and branches proposed in this paper, abstracting complex natural fracture networks into a series of basic topological units (I-shape, T-shape, X-shape) and their combinations, made systematic experimental research possible. It revealed a significant negative correlation between topological parameters (e.g., number of nodes, number of branches, normalized total fracture length) and the macroscopic breakdown pressure (Figure 8), indicating that topological structure can serve as an effective indicator for evaluating the overall resistance to hydraulic fracturing of fractured rock masses. This echoes the conclusions of Lei et al. [20] regarding numerical studies on fracture networks in terms of physical mechanisms; i.e., more complex topology usually implies higher initial damage and connectivity, thereby reducing the overall strength and fracture resistance of the rock mass.

The simplified method based on topological parameters introduced in Section 2.1 still has limitations. The Double T-upper and Double T-down specimens, which share identical topological parameters (number of endpoints, intersection nodes, branches, and normalized length), yielded equal average peak breakdown pressures in the experiments, despite observable discreteness in the individual peak pressure values from the three repeated tests. However, the T-X type specimen possesses more complex topological parameters than the Double T type, yet exhibited a slightly higher average peak breakdown pressure. Similarly, the 2T-X type specimen has increased topological parameters compared to the T-X type, but their average peak pressures are nearly identical (6.57 MPa vs. 6.54 MPa). This indicates that the simplified topological parameters alone cannot fully determine the fracturing characteristics. Using only these simple topological counting parameters to predict the fracture pressure of a specimen with a specific configuration still presents considerable uncertainty, as the wide 95% prediction band shown in Figure 8. Numerical simulation results confirm that crack initiation preferentially occurs at the tip with the most significant stress concentration, whose location is jointly governed by the topological configuration and boundary conditions. A more complex nodal arrangement may lead to dispersed stress concentration, thereby partially offsetting the strength-weakening effect induced by greater initial damage. Based on the findings of this study, future topological characterization methods may need to incorporate spatial orientation or configuration factors relative to the principal stress direction to more accurately predict hydraulic fracturing behavior.

Both experiments and simulations jointly indicate that hydraulic crack propagation is controlled by the synergy between the local stress field induced by the topological structure and the far-field principal stress direction. Figure 14 can intuitively explain the mechanical mechanism governing the initiation location strictly by “proximal-to-loading-end priority”. Under the interaction between the spatial position of the topological node and the far-field stress field, the degree of local stress concentration at the tip closest to the near loading end is much greater than at other tips. The final propagation path tends to follow the direction of the maximum principal stress, with the influence of fracture topology being insignificant. This indicates that the far-field stress has a stronger “guiding” effect on the crack propagation path. The breakdown pressure driving hydraulic fracturing is influenced by the stress difference under specific topological configurations. For example, the X-shaped node, by dispersing stress to multiple tips, has a higher breakdown pressure compared to the T-shaped node; although the T-X shape has a more complex topology, the stress dispersion effect introduced by its lower X-shaped node offsets the advantage of its greater initial damage, resulting in a slightly higher breakdown pressure.

The results of this study can provide a basis for optimizing perforation location design in fracturing operations. For instance, the “proximal-to-loading-end priority” initiation rule suggests that in fracturing design, perforation locations can be prioritized to target natural fracture nodes near the target layer boundary or stress concentration areas, thereby more effectively utilizing the topological structure to induce crack initiation and improve fracturing efficiency. Furthermore, the understanding of the influence mechanism of topological structure on hydraulic crack initiation and propagation can also enhance the level of fracturing design. For example, based on the dominant topological node types in the reservoir, designs can be targeted to induce the formation of more complex fracture networks.

### 5.2. Limitations and Future Perspectives

Although this study has obtained a series of valuable findings, there are still some limitations. This study was conducted only under uniaxial stress conditions, whereas rock masses in practical engineering are typically under true triaxial stress states. Confining pressure generally increases the fracture toughness and breakdown pressure of rock [49], and the constraint provided by the minimum horizontal principal stress strongly suppresses fracture deflection [50], favoring propagation along a plane perpendicular to it. In a three-dimensional stress field, the priority of crack initiation will be jointly determined by the spatial position of the topological node relative to the three principal stress directions, rather than solely by its distance from the loading boundary under uniaxial conditions. Furthermore, confining pressure may alter the nonlinear configuration-strengthening effect within the fracture network; we speculate that a reduced stress differential might diminish its influence. However, we believe the fundamental principle that topological structure influences competitive crack initiation by regulating local stress concentration remains valid, albeit with more complex governing criteria. The application of our conclusions to practical engineering must comprehensively consider the influence of the in situ stress field.

The use of a 2D model represents another simplification. The choice was made to enable direct comparison with our 2D plane-strain experiments and to manage the computational cost of 3D hydromechanically coupled DEM simulations. Consequently, this model cannot capture several key 3D effects: (1) the out-of-plane, tortuous propagation of fractures and the associated surface roughness, which influence fluid flow and shear behavior; (2) the full complexity of 3D stress shadow interactions between fractures; and (3) the true geometric intricacies of spatial fracture intersections. Despite these limitations, the core finding that topology governs initiation by modulating meso-scale stress concentration patterns is expected to be a transferable principle. However, quantitative predictions of breakdown pressure and exact fracture paths in real 3D settings require future dedicated investigation.

Future research work can focus on the following directions: developing more refined topological structure characterization methods that incorporate spatial orientation information; conducting further experimental or numerical simulation studies under true triaxial stress conditions; extending the investigation to three-dimensional fracture networks; considering the heterogeneity and anisotropy of rocks to be closer to the engineering geological characteristics of real reservoirs; and exploring the hydraulic crack propagation mechanisms under multi-field coupling conditions.

## 6. Conclusions

This paper, through systematic visual hydraulic fracturing tests and hydromechanically coupled discrete element numerical simulation, deeply studied the hydraulic crack propagation mechanisms in rock masses with topologically simplified fractures. The main conclusions are as follows:

(1) The initiation of hydraulic cracks in topological structure specimens strictly follows the “proximal-to-loading-end priority” rule. The interaction between the far-field stress and the local geometry maximizes the stress concentration and strain energy density at the tip nearest to the loaded boundary, thereby determining the preferential location for crack initiation.
(2) The breakdown pressure is governed by a dual, competing influence of fracture topology: a macroscopic weakening effect due to increased topological complexity versus a mesoscopic configuration-strengthening effect arising from dispersed stress concentration. While breakdown pressure generally exhibits a negative correlation with aggregate topological parameters (e.g., total number of nodes and branches), signifying an overall weakening due to elevated initial damage density, specific node configurations (e.g., X-shaped, T-X) can defy this trend. These configurations induce a “configuration-strengthening” effect by dispersing the applied load and stress concentration across multiple fracture tips, which can lead to a higher breakdown pressure than that of configurations with simpler topological parameters.
(3) Discrete element numerical simulation revealed the meso-mechanical mechanism by which topological structure influences crack propagation. The intensity and spatial distribution of stress concentration, engineered by the specific topological arrangement, fundamentally govern both the crack initiation location and the ultimate breakdown pressure. This finding shifts the perspective from a purely parametric description of topology (e.g., counting nodes) to a mechanistic understanding based on induced stress-field modulation.

In summary, this work establishes a critical link between the topological architecture of fracture networks and their hydro-mechanical response. It demonstrates that the hydraulic fracturing behavior is not solely dictated by the quantity of pre-existing fractures but is profoundly influenced by their topological configuration, which acts as a key mediator of internal stress distribution. These insights advance the fundamental understanding of fracture network mechanics and provide a mechanistic basis for optimizing fluid-driven fracturing strategies in engineered geo-materials and reservoirs by considering not just fracture density but also their topological signature.

## Figures and Tables

**Table 1:** Mechanical parameters of Befast granite [33].

Compressive Strength	Tensile Strength	Young’s Modulus	Poisson’s Ratio
127.94 MPa	6.49 MPa	77.16 GPa	0.23

**Table 2:** Number of nodes and branches for different topological structure specimens.

Category	Fracture Form	Endpoints	Intersection Nodes	Branches	Normalized Total Length
Simple Nodes	I	2	0	1	2
	Single T	3	1	3	4
	Single X	4	1	4	4
Mixed Nodes	T-X	5	2	6	7
	2T-X	6	3	8	9
Homogeneous Combination Nodes	Double T	4	2	5	6
	Double T Upper	4	2	5	5
	Double T Lower	4	2	5	5
	Double X	6	2	7	8
	3X	8	3	10	12

**Table 3:** Breakdown pressure of different topological structure specimens.

Category	Fracture Form	Breakdown Pressure/MPa			
		Group A	Group B	Group C	Average
Simple Nodes	I	7.27	7.95	9	8.07
	Single T	6.75	7.37	7.75	7.29
	Single X	7.27	8.43	8.72	8.14
Mixed Nodes	T-X	6.55	5.98	7.18	6.57
	2T-X	6.36	6.07	7.18	6.54
Combination Nodes	Double T	6.41	5.88	6.84	6.38
	Double T Upper	7.27	7.95	7.71	7.64
	Double T Lower	7.08	7.9	7.95	7.64
	Double X	6.5	5.98	5.69	6.06
	3X	5.59	5.5	5.59	5.56

**Table 4:** Meso-mechanical and hydraulic parameters for the model.

Parameter Category	Parameter Name	Symbol	Value
Meso-mechanical Parameters	Elastic Modulus	*E_f_*	80 GPa
	Stiffness Ratio	*κ_s_*	3.2
	Tensile Strength	*σ_t_*	25 MPa
	Friction Angle	*ϕ_s_*	48.9°
	Cohesive Strength	*c*	60 MPa
Hydraulic Interaction Parameters	Initial Channel Aperture	*a*_0_	0 μm
	Fluid Bulk Modulus	*κ*	2.2 GPa
	Fluid Viscosity	*μ*	1.0 × 10^−9^ MPa·s
	Injection Rate	*Q*	1 mm^3^/s

## Data Availability

The original contributions presented in this study are included in the article. Further inquiries can be directed to the corresponding author.

## Abbreviations

The following abbreviations are used in this manuscript:

DEM	Discrete Element Method
DIC	Digital Image Correlation
FEM	Finite Element Method
FJM	Flat-Joint Model
PFC	Particle Flow Code
SLZ	Strain Localization Zone
SLZs	Strain Localization Zones
XFEM	Extended Finite Element Method

## References

[1] Lei Q., Latham J.P., Tsang C.F. (2017). The use of discrete fracture networks for modelling coupled geomechanical and hydrological behaviour of fractured rocks. Comput. Geotech..

[2] Zhang X., Sanderson D.J. (1998). Numerical study of critical behaviour of deformation and permeability of fractured rock masses. Mar. Pet. Geol..

[3] Zhao C., Lei Q., Zhang Z. (2025). Impact of fracture networks on the structural deformation of lined rock caverns under high internal gas pressure. Undergr. Space.

[4] Zoback M.D., Rummel F., Jung R., Raleigh C.B. (1977). Laboratory hydraulic fracturing experiments in intact and pre-fractured rock. Int. J. Rock Mech. Min. Sci. Geomech. Abstr..

[5] Li B.Q., Einstein H.H. (2019). Direct and microseismic observations of hydraulic fracturing in Barre granite and Opalinus clayshale. J. Geophys. Res. Solid Earth.

[6] Liu X.W., Liu Q.S., Liu J.P., Kang Y.S., Li X.F. (2016). Experimental study on mechanism for fracture network initiation under complex stress conditions. Chin. J. Rock Mech. Eng..

[7] Zou Y., Zhang S., Zhou T., Zhou X., Guo T. (2016). Experimental investigation into hydraulic fracture network propagation in gas shales using CT scanning technology. Rock Mech. Rock Eng..

[8] Liu Z., Wang S., Zhao H., Wang W., Cheng Y. (2018). Effect of random natural fractures on hydraulic fracture propagation geometry in fractured carbonate rocks. Rock Mech. Rock Eng..

[9] Einstein H.H., Baecher G.B. (1983). Probabilistic and statistical methods in engineering geology: Specific methods and examples. Rock Mech. Rock Eng..

[10] Murray G.H. (1968). Quantitative fracture study—Sanish pool, McKenzie County, North Dakota. AAPG Bull..

[11] Kulatilake P.H.S.W., Fiedler R., Panda B.B. (1997). Box fractal dimension as a measure of statistical homogeneity of jointed rock masses. Eng. Geol..

[12] Sturzenegger M., Stead D. (2009). Close-range terrestrial digital photogrammetry and terrestrial laser scanning for discontinuity characterization on rock cuts. Eng. Geol..

[13] Jones R.R., Pearce M.A., Jacquemyn C., Watson F.E. (2016). Robust best-fit planes from geospatial data. Geosphere.

[14] Wu H., Pollard D.D. (2002). Imaging 3-D fracture networks around boreholes. AAPG Bull..

[15] Dershowitz W.S., Einstein H.H. (1988). Characterizing rock joint geometry with joint system models. Rock Mech. Rock Eng..

[16] Bonnet E., Bour O., Odling N.E., Davy P., Main I., Cowie P., Berkowitz B. (2001). Scaling of fracture systems in geological media. Rev. Geophys..

[17] Allegre C.J., Le Mouel J.L., Provost A. (1982). Scaling rules in rock fracture and possible implications for earthquake prediction. Nature.

[18] Zhou Z., Su Y., Wang W., Zhang Q. (2017). Application of the fractal geometry theory on fracture network simulation. J. Pet. Explor. Prod. Technol..

[19] Bour O., Davy P., Darcel C., Odling N.E. (2002). A statistical scaling model for fracture network geometry, with validation on a multiscale mapping of a joint network (Hornelen Basin, Norway). J. Geophys. Res. Solid Earth.

[20] Lei Q., Latham J.P., Tsang C.F., Xiang J., Lang P. (2015). A new approach to upscaling fracture network models while preserving geostatistical and geomechanical characteristics. J. Geophys. Res. Solid Earth.

[21] Lei Q., Wang X., Min K.B., Xiang J., Lan B. (2020). Interactive roles of geometrical distribution and geomechanical deformation of fracture networks in fluid flow through fractured geological media. J. Rock Mech. Geotech. Eng..

[22] Sanderson D.J., Nixon C.W. (2015). The use of topology in fracture network characterization. J. Struct. Geol..

[23] Morley C.K., Nixon C.W. (2016). Topological characteristics of simple and complex normal fault networks. J. Struct. Geol..

[24] Peacock D.C.P., Nixon C.W., Rotevatn A., Sanderson D.J., Zuluaga L.F. (2016). Glossary of fault and other fracture networks. J. Struct. Geol..

[25] Duffy O.B., Nixon C.W., Bell R.E., Sanderson D.J., Armstrong J. (2017). The topology of evolving rift fault networks: Single-phase vs multi-phase rifts. J. Struct. Geol..

[26] Santiago E., Velasco-Hernández J.X., Romero-Salcedo M. (2016). A descriptive study of fracture networks in rocks using complex network metrics. Comput. Geosci..

[27] Sanderson D.J., Peacock D.C.P., Nixon C.W., Rotevatn A. (2019). Graph theory and the analysis of fracture networks. J. Struct. Geol..

[28] Zhu W., Khirevich S., Patzek T.W. (2021). Impact of fracture geometry and topology on the connectivity and flow properties of stochastic fracture networks. Water Resour. Res..

[29] Sanderson D.J., Nixon C.W. (2018). Topology, connectivity and percolation in fracture networks. J. Struct. Geol..

[30] Li B.Q., Da Silva B.G., Einstein H.H. (2019). Laboratory hydraulic fracturing of granite: Acoustic emission observations and interpretation. Eng. Fract. Mech..

[31] Ishida T., Aoyagi K., Niwa T., Chen Y., Murata S., Chen Q., Nakayama Y. (2012). Acoustic emission monitoring of hydraulic fracturing laboratory experiment with supercritical and liquid CO_2_. Geophys. Res. Lett..

[32] Zhao C., Xing J., Zhou Y., Gong X., Zhang R. (2020). Experimental investigation on hydraulic fracturing of granite specimens with double flaws based on DIC. Eng. Geol..

[33] Yu J., Li N., Hui B., Sun H., Zhang R., Chen X. (2024). Experimental simulation of fracture propagation and extension in hydraulic fracturing: A state-of-the-art review. Fuel.

[34] Fu H., Huang L., Hou B., Chen M., Chen W., Chen Y. (2024). Experimental and numerical investigation on interaction mechanism between hydraulic fracture and natural fracture. Rock Mech. Rock Eng..

[35] Huang L., Li B., Wang B., Li X., Li J. (2023). Effects of coal bedding dip angle on hydraulic fracturing crack propagation. Geomech. Geophys. Geo-Energy Geo-Resour..

[36] Silva B.G., Einstein H.H. (2018). Physical processes involved in the laboratory hydraulic fracturing of granite: Visual observations and interpretation. Eng. Fract. Mech..

[37] Fan T.G., Zhang G.Q. (2014). Laboratory investigation of hydraulic fracture networks in formations with continuous orthogonal fractures. Energy.

[38] Liu X., Liu Q., Liu B., Zhu Y. (2019). Failure behavior for rocklike material with cross crack under biaxial compression. J. Mater. Civ. Eng..

[39] Potyondy D.O., Cundall P.A. (2004). A bonded-particle model for rock. Int. J. Rock Mech. Min. Sci..

[40] Zhang X.P., Wong L.N.Y. (2012). Cracking processes in rock-like material containing a single flaw under uniaxial compression: A numerical study based on parallel bonded-particle model approach. Rock Mech. Rock Eng..

[41] Zhang R., Zhao C., Yang C., Zhou Y., Xing J. (2021). A comprehensive study of single-flawed granite hydraulically fracturing with laboratory experiments and flat-jointed bonded particle modeling. Comput. Geotech..

[42] Ehlers W., Luo C. (2017). A phase-field approach embedded in the theory of porous media for the description of dynamic hydraulic fracturing. Comput. Methods Appl. Mech. Eng..

[43] Xing J., Zhao C. (2023). A hydro-mechanical phase field model for hydraulically induced fractures in poroelastic media. Comput. Geotech..

[44] Xing J., Zhao C., Xiang F., Niu J., Chen H., Zhou B., Zhou Y. (2025). A Hydro-Mechanical Phase Field Model for Hydraulic Fracturing of Grain-Structured Rocks Based on Voronoi Tessellation. Rock Mech. Rock Eng..

[45] Xing J., Zhao C., Huang L., Zhou Y., Zhang R. (2021). Direct observations of hydraulic fracturing in rock bridge of granite specimens in grain-scale. IOP Conf. Ser. Earth Environ. Sci..

[46] Gong X., Zhao C., Chen H., Xing J., Ni J. (2025). Crack Evolution and Mechanical Behavior of Granite with Topological Flaws Under Uniaxial Compression. Geotech. Geol. Eng..

[47] Tian Y., Zhao C., Xing J., Gong X., Ni J. (2024). A new digital image correlation method for discontinuous measurement in fracture analysis. Theor. Appl. Fract. Mech..

[48] Blaber J., Adair B., Antoniou A. (2015). Ncorr: Open-source 2D digital image correlation matlab software. Exp. Mech..

[49] Zhou Q., Xie H.P., Zhu Z.M., He R., Lu H.J., Fan Z.D., Nie X.F., Ren L. (2023). Fracture toughness anisotropy in shale under deep in situ stress conditions. Rock Mech. Rock Eng..

[50] Warpinski N.R., Teufel L.W. (1987). Influence of geologic discontinuities on hydraulic fracture propagation (includes associated papers 17011 and 17074). J. Pet. Technol..

